# Engineering plants with carbon nanotubes: a sustainable agriculture approach

**DOI:** 10.1186/s12951-022-01483-w

**Published:** 2022-06-14

**Authors:** Mahpara Safdar, Woochan Kim, Sunho Park, Yonghyun Gwon, Yeon-Ok Kim, Jangho Kim

**Affiliations:** 1 Department of Convergence Biosystems Engineering, Chonnam National University, Gwangju, 61186 Republic of Korea; 2 Department of Rural and Biosystems Engineering, Chonnam National University, Gwangju, 61186 Republic of Korea; 3 Interdisciplinary Program in IT-Bio Convergence System, Chonnam National University, Gwangju, 61186 Republic of Korea

**Keywords:** Carbon nanotubes, Agriculture, Sustainability, Plant growth, Antimicrobial activity, Gene delivery, Biosensors, Environmental stress

## Abstract

Sustainable agriculture is an important conception to meet the growing food demand of the global population. The increased need for adequate and safe food, as well as the ongoing ecological destruction associated with conventional agriculture practices are key global challenges. Nanomaterials are being developed in the agriculture sector to improve the growth and protection of crops. Among the various engineered nanomaterials, carbon nanotubes (CNTs) are one of the most promising carbon-based nanomaterials owing to their attractive physiochemical properties such as small size, high surface area, and superior mechanical and thermal strength, offering better opportunities for agriculture sector applications. This review provides basic information about CNTs, including their history; classification; and electrical, thermal, and mechanical properties, with a focus on their applications in the agriculture field. Furthermore, the mechanisms of the uptake and translocation of CNTs in plants and their defense mechanisms against environmental stresses are discussed. Finally, the major shortcomings, threats, and challenges of CNTs are assessed to provide a broad and clear view of the potential and future directions for CNT-based agriculture applications to achieve the goal of sustainability.

## Introduction

Currently, the agriculture sector is facing a wide range of challenges such as climate change, plant pathogenic organisms, infectious crop diseases, soil nutrient deficiency, crop yield reduction, lack of awareness of genetically modified crops, and a lack of workforce, which threaten to destabilize agriculture sustainability [1–3]. As the climate change conditions continue to develop, more extreme environmental conditions are becoming more frequent, such as salinity, drought, high and low temperatures, which can cause extensive annually reductions in overall crop production, yield, and quality worldwide [4, 5]. Abiotic stresses, particularly drought and high temperatures, have increases in frequency and intensity as a result of climate change, resulting in significant losses in major cereal crops such as wheat, maize, and barely. For instance, drought and soil salinity in high levels, as well as their secondary effects such as osmotic, oxidative, and ionic stress, are recognized to be major obstacles to agricultural output [6–9]. Furthermore, biotic stresses such as pests and diseases reduced the agriculture production by approximately 20–30% annually, which is considered as the most discouraging challenge to achieving food security globally [10, 11].

In previous decades, several alternative approaches have been deployed to promote agricultural sustainability, such as chemical inputs (commercial fertilizers and pesticides), crop rotation, precision farming, urban farming and genetic modifications through targeted breeding and gene manipulation. Several conventional and molecular approaches also have been used in crop breeding, including functional genomic tools, genetic selection, mutagenic breeding, physical maps, soma clonal variations, and whole-genome sequence-based approaches [12]. In fact, genetic engineering varieties are only available for a small portion of food crops such as maize, soybean, canola, rice, potato, papaya, squash, and apple that uses modern biotechnology tools to introduce, eliminate, or rearrange specific genes. By contrast, commercial conventional breeding releases hundreds of new crop types each year to improve crop production, food security, nutrition, and customer choice. Conventional plant breeding involves identifying parent plants with desirable characteristics to create favorable combinations in the next generation [13]. These methods have been developed to increase commercially valuable traits in plants, including agriculture productivity, by enhancing stress tolerance and breeding such crops with various stress-tolerant traits. However, existing technologies have some major drawbacks, including limited availability of specific crop genes, low success rates, significant ecological consequences, time-consuming processes, and public concerns about genetically modified crops [14, 15]. Chemical priming is another inspiring alternative approach that can help crops resist environmental stresses through establishing defense pathways without employing genetic changes [16]. To overcome these problems, farmers have adopted the practice of excessive utilization of agrochemicals to manage these losses and enhance crop yields [17]. However, the excessive use of conventional fertilizers for a long period leads to severe environmental problems such as air pollution, soil quality degradation, water eutrophication, and groundwater pollution [18–20]. In addition, chemical fertilizers have low efficiency due to volatilization and leaching, which cause environmental contamination and raise production costs, posing a constraint on achieving adequate agricultural sustainability [21]. Therefore, new approaches that can improve the efficiency in the utilization of agrochemicals while effectively protecting crops from environmental stresses are required to fulfill current and future food demands safely and sustainably [22–25].

Nanotechnology is a promising candidate for sustainable agriculture, which is expected to transform conventional farming into precision farming. Precision farming is a well-balanced strategy for increasing agriculture yields by monitoring environmental variables and implementing precisely controlled actions in response to each environmental condition [26]. Agriculture is a highly complex activity and used to be ungrateful with revolutions and drastic approaches such as the use of genetically modified crops and agrochemicals. Nanotechnology has the potential by improving crop yields while maintaining ecological balance, environmental sustainability, and economic stability [2, 3]. Several researchers have recently focused on nanotechnology-based agriculture to increase agricultural productivity through efficient nutrient delivery, nutrient control, reducing mobile nutrient losses, developing slow-release fertilizers, and improving the accessibility of poorly available nutrients [23, 27–29].

NMs are considered an ideal platform for leading the agri-nanotech revolution owing to their advantages of an incredibly small size (< 100 nm), allowing them to pass through biological barriers and permeate plant tissues via foliar or root application, therefore providing novel and efficient routes for nutrients and pesticide delivery [30, 31]. The most effective applications of NMs in agriculture involve the use of nanofertilizers, which improve growth and crop productivity; the suppression of plant diseases; and nanosensors to monitor soil quality and plant health [32–35]. Compared with particulate matter or bulk particles, the use of NMs shows great efficiency due to their diverse functions, large surface area, high stability, the presence of active sites on the surface, and high adsorption capacity [36, 37]. For agriculture applications, NMs can deliver herbicides, fertilizers, and pesticides over a larger specific surface area and ensure their “on-demand” release, whether the goal is to prevent pathogens, pests, and diseases, or to meet nutritional requirements. Thus, NMs can promote nutrient delivery, resulting in improved crop growth, yield, and quality [38–41].

NM engineering has emerged as a promising technology for sustainably improving the efficiency of present agricultural practices and overall crop productivity [42]. Among the various types of carbon-based nanomaterials (CBNs), carbon nanotubes (CNTs) have attracted more attention in agricultural applications owing to their impacts on plant growth regulation, ability to cross plant cell walls, agricultural smart delivery, nano transport, and as a medium for biosensors [43–46].

CNTs are considered a novel fertilizer given their capacity to function as both a slow-release fertilizer and a plant growth booster [47, 48]. CNTs can be used in agriculture as smart delivery systems for nano-encapsulated agrochemicals that are time-controlled, self-regulated, and specifically targeted for components such as fertilizers and pesticides [48–50]. Nano-carbon fertilizer is one of the applications of CNT in agriculture. Nano-carbon may adsorb nitrogen from ammonia and release hydrogen ions, allowing plants to absorb more water and nutrients. As a result, the plant’s uptake of N, P, and K would be improved. It was investigated by field experiment that applying nitrogen and nano-carbon together could boost the output and quality of rice. The results also revealed that nano-carbon might be employed as a coating material for slow-release fertilizers and incorporation of nano-carbon into slow-release fertilizer could help to reduce water pollution [51, 52]. Effective delivery of nutrients is highly dependent on plant water uptake. Due to their high water conductivity, CNTs can be developed from a variety of NMs and are ideally suited as a nanoplatform for assessing and treating diverse nutritional antagonisms [53, 54]. Agrochemicals or other compounds can be delivered to hosts using CNT-based delivery systems, resulting in fewer chemicals being discharged into the environment and less damage to other plant tissues [55, 56].

Plant–CNT complexes alter the physiological and morphological characteristics of plants depending on the size, concentration, solubility, and type of CNTs, as well as the particular plant species and plant growth stages. The uptake and translocation of CNTs in plants have been investigated by several researchers who have proposed a strategy based on the ratio of CNT size to cell wall pore size [57, 58]. Ultrasonic-assisted chemical oxidative cutting and the addition of carboxylic groups to CNTs increase their solubility and uptake into plants [59, 60]. CNT uptake into plants has been investigated under various plant growth conditions such as with agar medium and hydroponic culture, providing contradictory results [58, 61, 62]. Another pilot-scale study was conducted to elucidate the toxicity effects of functionalized and non-functionalized single-walled carbon nanotubes (SWCNTs) on root elongation of various crops such as carrot, cabbage, tomato, onion, and lettuce. Both SWCNTs and f-SWCNTs affected root elongation in all selected crops. However, phytotoxicity varied between types of SWCNTs, with SWCNTs affecting more species. Tomato and lettuce were the most sensitive species, with highly significant reductions in root length observed at 24 and 48 h after exposure to CNTs. In onions and cucumbers, non-functionalized CNTs increased root elongation. In addition, root elongation of cabbage and carrot was not affected by the presence of either f-CNTs or CNTs. Effects observed following exposure to f-CNTs or CNTs were more pronounced at 24 h compared at 48 h [63].

Several studies have recently demonstrated that a low concentration of CNTs improves seed germination and seedling growth in tomato, soybean, and corn. The effects of CNTs on plant growth and reproduction can be induced directly by the interactions of CNTs with plant proteins, transcription factors, or DNA, or indirectly by inducing an increase in nitric oxide and reactive oxygen species (ROS) production [64]. Additionally, CNTs were shown to increase the chlorophyll content and photosynthesis, resulting in the promotion of the plant’s vegetative growth. Furthermore, under salt-stressed conditions, CNTs also caused alterations in the lipid content, flexibility, and permeability of the root plasma membranes, resulting in enhanced aquaporin transduction [65]. This property of CNTs could be beneficial in maximizing water consumption in plants, especially in areas of water scarcity [66].

In this review, we provide a detailed history and a broad overview of the applications of CNTs in the agriculture field. We particularly aimed to provide up-to-date information and a complete mechanistic understanding of the new paradigms of the applications and progress of CNTs in agriculture by investigating their roles in promoting seed germination and plant growth, antibacterial activity, gene delivery, and as nanosensors, toward designing a better and more sustainable agriculture system for the future. Moreover, we discuss the mechanism by which CNTs can promote sustainable agriculture through their uptake, translocation, accumulation, and defense mechanisms against environmental stresses. Finally, CNT-related challenges and future perspectives in the agriculture field are highlighted.

## Classification of CNTs

**Fig. 1:**
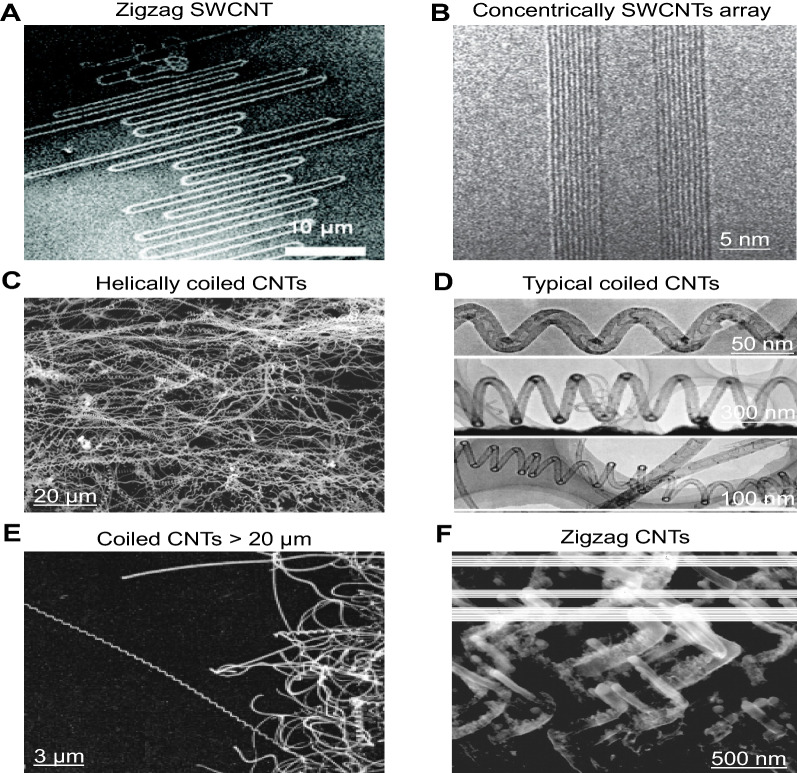
Schematic illustration of the structure and morphology of CNTs. **A** Scanning electron microscope (SEM) image of a zigzag shaped SWCNT [74]. **B** Transmission electron microscope (TEM) image of a MWNT containing a concentrically nested array of nine SWNTs [75]. **C** SEM image of large amount of helically coiled CNTs [76]. **D** TEM images of typical coiled CNTs with a nanobell structure [77]. **E** SEM images of the coiled CNTs > 20 mm in length [77]. **F** SEM image of array of CNTs grown with zigzag morphology using a three-stage growth process [78]

In recent decades, CNTs have been considered among the most important tools in various fields. A CNT is a versatile allotrope form of carbon with a cylindrical, long, tubular structure comprising rolled-up graphene sheets. CNTs can take on various structures depending on the length, thickness, and number of layers [67]. CNTs typically have a diameter ranging from 1 nm up to 50 nm. Normally, they are several microns long, although recent advancements have enabled construction of much longer nanotubes in the centimeter scale. A graphene sheet can be used to synthesize different types of nanotubes by rolling the tubes down in various configurations [68]. CNTs can be classified into two main types based on their structures: SWCNTs comprise a single layer of a graphene sheet with diameters ranging from 0.4 to 2 nm, whereas multi-walled carbon nanotube (MWCNTs) consists of multilayer graphene sheets with outer and inner diameters of 2–100 nm and 1–3 nm, respectively, and with lengths ranging from 0.2 nm to several microns. CNTs can take on unique structures, such as hollow graphite cylinders with hexagonally organized carbon rings, [68] and have high tensile strength (~ 200 Gpa), which is similar to that of graphene, but are more stable than graphene, even at extremely high temperatures, and optimize vibrational entropy [69]. Due to the presence of multiple layers of carbon atoms, MWCNTs have higher mechanical strength as compared with SWCNTs. CNTs also have a stronger young’s modulus and greater tensile strength than metals such as steel and iron [70–73]. SWCNTs form crystal-like structures when hexagonally organized in a bundle, which can be classified into numerous types based on the wrapping style, chirality, zigzag, and armchair form. In Fig. 1A, scanning electron microscopy (SEM) describe that the unusual zigzag shape which is mainly due to SWCNT-substrate lattice interaction and gas flow [74]. MWNTs comprise an array of such nanotubes that are concentrically nested like rings of a tree trunk, is one of main types of CNTs that can have high structural perfection (Fig. 1B) [75]. Coiled CNTs produced by catalytic chemical vapor deposition (CVD) on an iron-coated indium tin oxide substrate. CNTs are found in helical forms more than 95% of cases. Each Coil grows with its own diameter and pitch as shown in Fig. 1C [76]. Transmission electron microscopy (TEM) is used to analyze the structure of the coiled CNTs. Similar to the straight CNTs, coiled CNTs are built in a series of short tubes along the tube axis. The majority of the short tubes are bell-shaped, with one end capped and the other open, and are referred to as “nanobells” as shown in Fig. 1D [77]. SEM image shows that a coiled CNT in a length of 20 mm with regular pitch extends out from the template in Fig. 1E [77]. The zigzag morphologies consist of very sharp and alternating ∼90° bends. They were grown using a plasma enhanced CVD process. The bending of the CNTs during growth was caused by changing the direction of the electric field lines in the growth region of the sample (Fig. 1F) [78]. SWCNTs have nearly identical properties to MWCNTs, with the exception of their higher tensile strength [79, 80].

## Structural, electrical, thermal, and mechanical properties of CNTs

CNTs have exceptional structural, electrical, thermal, and mechanical properties and can be used alone or in combination to create sensitive sensors or multifunctional materials [81, 82]. Several researchers have demonstrated that CNTs exhibit unique conductive properties. These findings were the first to suggest that geometrical changes such as defects, chirality, diameter, and the degree of crystallinity of the tubular structure, have a significant impact on the electrical properties of CNTs [83, 84]. The diameter and helicity of graphene influence the semiconductor or metallic potential of CNTs [85, 86]. Metal SWCNTs in rope form have a resistance of roughly 10^− 4^ Ω cm at 300 K [87]. Because of the presence of sp^2^ hybridized covalent bonds, CNTs exhibit greater thermal conductivity than sp^3^ hybridized diamonds [88, 89]. The temperature and phonon mean-free path widely influence the thermal conductivity of CNTs. At room temperature, the thermal conductivity of SWCNTs is in the range of 1800–6000 W/m·K, whereas MWCNTs have a thermal conductivity of approximately 3000 W/m·K. The thermal properties of CNTs are also influenced by their functionalization [90, 91]. The mechanical strength of CNTs is mainly due to their strong sp^2^ covalent bonds. CNTs with diameters ranging from 1.0 to 1.5 nm have an average Young’s modulus value of ~ 1.25 TPa, which is higher than the in-plane modulus value of graphite [92, 93]. The chirality and diameter of CNTs strongly influence the elastic properties of SWCNTs [94, 95].

The procedures that are commonly available for the synthesis of CNTs include chemical vapor deposition (CVD), [96–99] arc discharge, [83, 100–102] and laser ablation (LA) [103, 104]. Earlier arc discharge and laser ablation techniques generally required a high temperature of approximately  > 1700 °C during synthesis. However, these techniques were eventually supplanted by chemical vapor deposition, which requires a lower temperature (800 °C), resulting in a well-aligned structure and desired layer orientation [105]. CVD is a class of methods that appear to give the best prospect of obtaining a regulated process for the selective synthesis of nanotubes with predefined features. The CVD process entails the catalyst-assisted breakdown of hydrocarbons, commonly ethylene or acetylene, in a tube reactor at 550 − 750 °C, followed by the development of CNTs over the catalyst as the system cools. The working parameters, such as temperature and operation pressure, the type, volume, and concentration of hydrocarbon, the nature, size, and pretreatment of metallic catalyst, the nature of the support, and the reaction duration, all influence the features of carbon nanotubes produced by CVD [106, 107]. Arc discharge is the oldest and most common technique to produce large quantities of unpurified nanotubes, much effort is being put into developing production processes that give more controllable nanotube synthesis routes. This approach relies on the electrical breakdown of a gas to generate plasma. In compared to other approaches, it uses greater temperatures (over 1700 °C) to evaporate carbon atoms in plasma, resulting in CNT development with minimal structural flaws [68, 108]. laser ablation (LA) is one of the best ways for producing SWNTs. LA offers the benefit of creating high-quality, high-yield, and high-purity SWNTs in a short amount of time: 500 mg of SWCNTs in 5 min with up to 90% purity. However, this method is not interesting for the synthesis of MWNTs because of its expensive cost [109, 110].

Non-standard methods such as pyrolysis and hydrothermal treatment have also been used for CNT synthesis in addition to these well-established techniques [111]. Because of the presence of several undesirable byproducts such as carbonaceous residues, amorphous carbon, fullerenes, and catalyst impurities, the synthesized CNTs are impure. This led scientists to focus their efforts on purifying manufactured CNTs. Synthesizing high-purity CNTs, particularly to achieve specific lengths and diameters, remains a challenge in this current era of extremely inventive technology-driven procedures [112–114]. Owing to their small size, highly advanced techniques are required to assess their characteristics and morphology. Moreover, because of their bundled and aggregated structure, the usage of CNTs is quite difficult. CNTs must be disseminated in water or another solvent for application purposes to improve their properties. This can be accomplished via a combination of centrifugation, sonication, oxidation, and ultrasonication, as well as with the use of dispersing agents. To determine the concentration of dissolved CNTs in a solvent, characterization must be performed. The techniques that are typically used to characterize the morphology, texture, and structure of CNTs are divided into four categories: spectroscopic, [115–117] thermal, [118, 119] microscopic, and diffraction procedures [120, 121]. Raman spectroscopy (RS) is one of the most powerful characterization technique for CNTs. It is routinely employed to evaluate the quality and purity of as-prepared CNTs. CNTs have two main first order bands such as D and G band in their Raman spectrum. The D band is correlated with defects in CNTs, which is observed at roughly 1300–1350 cm^− 1^ whereas, G band corresponds to the degree of graphitization of carbon nanotubes, which is roughly 1500–1600 cm^− 1^. As a result, the area ratio of the D and G bands (ID /IG) is commonly used to determine the defect level in a CNTs sample [122, 123]. Scanning electron microscopy (SEM) and transmission electron microscopy (TEM) are the well-known techniques that are commonly used to observe the position of tip and sidewall, as well as the morphology of CNTs [124, 125]. Infrared spectroscopy, X-ray photoelectron spectroscopy (XPS) and thermogravimetric analysis (TG) are usually used to precisely verify the occurrence of functionalization reactions of CNTs for final quality evaluation [126, 127].

## History and possible applications of CNTs

CNTs are a relatively recent NM, which have only been widely recognized and studied in the last decade. CNTs were first discovered and characterized in 1952 by Radushkevich and Lukyanovich [128] and single-walled carbon nanotubes (SWCNTs) were subsequently discovered and described by Oberlin et al. [129] in 1976. However, in most of the recent historical literature, Iijima is credited with the discovery of CNTs, as the first scientist to describe the multi-walled carbon nanotube (MWCNT) preparation process in 1991 [83]. The unique properties of CNTs, including their large surface area, small size, and reactivity, provide excellent opportunities for their use in the agriculture sector. In 2008, Cañas et al. [63]. demonstrated the effect of functionalized and non-functionalized SWCNTs on root elongation in several crops such as tomato, onion, cucumber, lettuce, cabbage, and carrot. Seeds of different plant species were exposed to both types of CNTs in petri dishes and placed in a growth chamber at 25 °C. Non-functionalized CNTs had a greater impact on root length than functionalized CNTs. Tomato root elongation was inhibited by non-functionalized nanotubes, whereas onion and cucumber root elongation were improved. Functionalized nanotubes reduced root elongation in lettuce, whereas cabbage and carrots were unaffected by any type of SWCNT [63]. Lin et al. [43] reported that CNTs penetrated the plant cell wall and membrane, which can be used to develop smart delivery systems in plants. Confocal fluorescence images indicated the cellular absorption of SWCNT–fluorescein isothiocyanate (FITC) and SWCNT–DNA conjugates, confirming the potential of SWCNTs as nanotransporters to pass through plant cell walls. The authors further demonstrated that SWCNTs have the ability to carry various cargoes to different plant cell organelles [43]. Similarly, CNTs were reported to penetrate the thick seed coat and enhance the water uptake process, which could contribute to rapid seed germination and stimulate early growth in young seedlings [130].

**Fig. 2:**
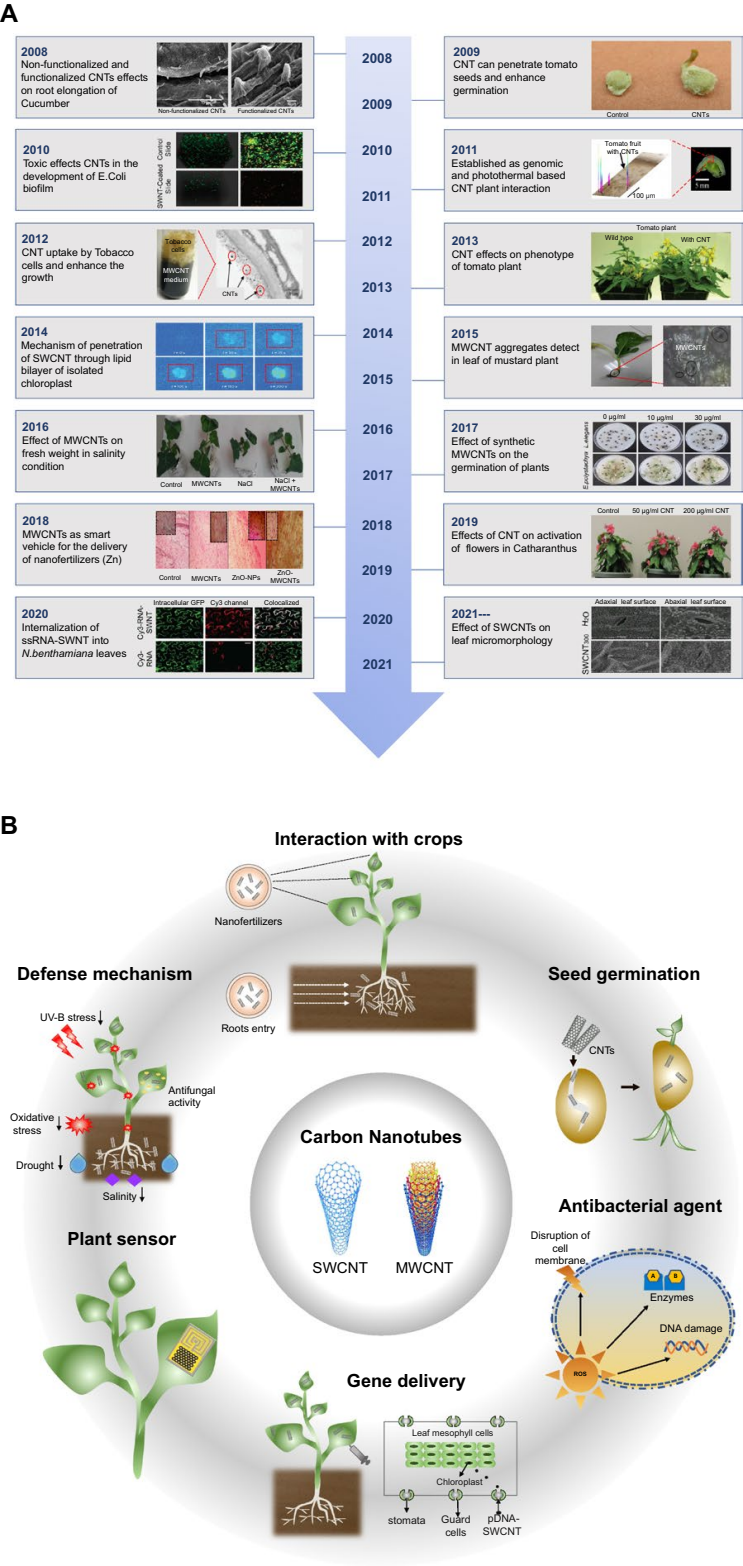
Introduction of carbon nanotubes (CNTs). **A** Timeline of CNT development for applications in the agriculture sector to improve plant growth. CNTs were first discovered by Iijima in 1991 and were first introduced in agriculture in 2008. For sustainable agriculture production, CNTs improve seed germination and growth, exhibit antimicrobial activity, can be used in gene delivery and as biosensors, and protect plants against various environmental stresses. **B** Diverse applications of CNTs in the agriculture field

In early studies, CNTs were recognized as promoters of seed germination and growth of crops [60, 130–132]. Moreover, researchers demonstrated the impact of SWCNTs as an antibacterial agent, as they can inhibit the growth and development of microbial biofilms [133]. CNTs doubled the flower and fruit production compared with that of the control by changing the composition of soil microbiota [53]. CNTs have also attracted attention given their ability to reduce the salinity stress of crops. MWCNTs were found to accumulate at higher concentrations in plant cells under salinity conditions and could enhance the transduction of aquaporin, which can improve water uptake and transport to reduce the adverse effects of salinity stress [54]. Another study demonstrated that CNTs are responsible for the influence of reproductive growth in fiber-producing crops and ornamental species, as highlighted by the significant acceleration in total flower production in cotton and *Catharanthus* by 37% and 58%, respectively [134]. Furthermore, CNTs have been applied in gene delivery [135] and as nanosensors [32]. The detailed history of CNTs in the agriculture field is summarized in Fig. 2A, and the possible applications of CNTs in the agriculture field are summarized in Fig. 2B.

## Effects of CNTs during the life cycle of plants

NMs are informed to improve crop growth and development from initial phase of seed germination to death. The application of NMs to overall growth and development process in crops is mainly dependent on size, concentration, physical, and chemical features. CNTs have attracted substantial interest in the agricultural sector owing to their possible applications in enhancing plant growth and seed germination, their ability to penetrate plant cell walls, and for cellular delivery. CNTs have attracted substantial interest in the agricultural sector owing to their possible applications in enhancing plant growth and seed germination, their ability to penetrate plant cell walls, and for cellular delivery [136–138].

### Effects on seed germination

**Fig. 3:**
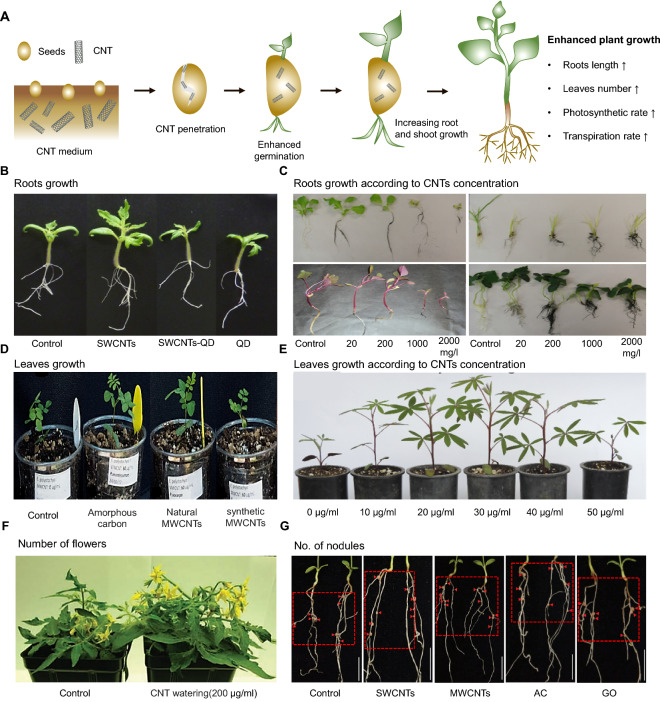
Effects of CNTs during the life cycle of plants. **A** Schematic representation of the effects of CNTs at various stages of plant growth. **B** The effect of single-walled carbon nanotubes (SWCNTs), quantum dots (QDs), and SWCNT-QD conjugates on the phenotype of 2-month-old tomato plants grown on a medium supplemented with 0.5 µg/mL QDs, 50 µg/mL SWCNTs, or 50 µg/mL SWCNT-QDs, or without nanoparticles as a control [144]. **C** Morphological observations of red spinach and lettuce exposed to multi-walled carbon nanotubes (MWCNTs) at concentrations of 0, 20, 200, 1000, and 2000 mg/L in hydroponic culture for 15 days [146]. **D** Image showing the effects of natural MWCNTs, synthetic MWCNTs, amorphous carbon, and a control group on *Eysenhardtia polystachya* growth [141]. **E** Effect of synthetic MWCNTs on the growth of *Lupinus elegans* [142]. **F** Effect of water supplied CNTs (50 and 200 µg/mL) on the number of flowers on tomato plants [53]. **G** Effect of CNTs on the nodule development of plants grown in soil treated with activated carbon (AC), MWCNTs, SWCNTs, and graphene oxide (GO) at low (50 µg/mL) or high (500 µg/mL) concentration for 14 days post-inoculation (dpi), respectively. Nodules on the roots are shown by red triangles. Scale bar = 10 mm [147]

Seed germination is the first step and the most sensitive period in the life cycle of plants. Numerous studies have demonstrated that the use of nanotechnology has beneficial effects on seed germination. For instance, Khodakovskaya et al. [130] demonstrated that CNTs can penetrate tomato seed coats, leading to acceleration in the seed germination rate and seedling growth on medium containing 10−40 µg/mL of CNTs (Fig. 3A). They further observed that tomato seeds in the presence of CNTs germinated and grew more quickly than those in the absence of CNTs because CNTs accelerated water uptake mechanisms by penetrating the seed coats: in 20 days, the MWCNTs improved seed germination by up to 90% as compared to an increase of 71% in the control group and improved plant biomass by up to 50%. Mondal et al. [139] also found that MWCNTs improved seed germination and plant growth in mustard plants. They discovered that oxidized MWCNTs were more effective at lower concentrations (2.3 × 10^− 3^ mg/mL) than non-oxidized MWCNTs based on the germination index and relative root elongation. In addition, Lahiani et al. [140] determined the effects of MWCNT (50, 100, and 200 g/mL) application in agar media on the germination and growth of soybean, corn, and barley after 10–11 days. In comparison to the untreated group, the germination rate was increased by approximately 50% in barley and soybean and by approximately 90% in corn after exposure. Similarly, the shoot (corn) and root (soybean) lengths were also enhanced by approximately 40% and 26%, respectively. Moreover, natural MWCNTs obtained after a wildfire were found to promote the germination and growth of *Eysenhardtia polystachya* as compared to those observed following exposure to amorphous carbon synthetic MWCNTs. Natural MWCNTs increased the fresh and dry biomass, particularly at 40 g/mL, and further increased the leaf number, root growth, and dry and fresh weights of the seedling shoots and roots (Fig. 3D) [141]. A similar effect of MWCNTs obtained after natural forest fires was demonstrated in *Lupinus elegans*; the germination and biomass of *L. elegans* were considerably increased with exposure to 30 g/L MWCNTs, although there was a significant decline with exposure to 50 g/L MWCNTs (Fig. 3E) [142]. CNTs were also found to influence the germination of tomato, onion, turnip, and radish seeds at four concentrations of 0, 10, 20, and 40 mg/L. Tomato and onion germination were improved by the CNTs at 10–40 mg/L to a larger extent than that for the radish and turnip [60].

### Effects on physiological and morphological response

NPs can influence the growth and yield of crops by changing the morphology and physiology of plants. Cañas et al.[63] examined the effects of functionalized and non-functionalized CNTs on the root elongation of six selected crops (cabbage, carrot, cucumber, lettuce, onion, and tomato) and observed that non-functionalized CNTs enhanced the length of roots in the onion and cucumber, whereas the CNTs were not able to penetrate the roots. Tiwari et al. [143] studied the effects of MWCNTs on tomato seedlings and discovered that absorption of vital nutrients increased at concentrations of 40 g/mL MWCNTs, which consequently improved plant growth and biomass. Another study revealed that the addition of SWCNTs enhanced tomato seedling growth. However, the exposure of tomato seedlings to SWCNTs along with quantum dots (50 µg/mL) significantly reduced the chlorophyll content by 1.5-fold in leaves, and the total weight and height of the tomato root/shoot system decreased by four times compared with those in the control group (Fig. 3B) [144]. Likewise, another study demonstrated that pure MWCNTs (20 mg/L) could increase the uptake of water and nutrients in maize plants, resulting in increased biomass of the plant. The interaction of the MWCNT (at 20 mg/L) with the + 2 and + 3 oxidation states of Fe in connection to Fe supply as nutrition to seedlings in agarose media was examined using polarized energy dispersive X-ray fluorescence analysis (pEDXRF) spectrometry. This feature of CNTs was considered to have a beneficial effect on plant growth in nutrient-deficient soil [145]. In 2012, Khodakovskaya et al. [131] showed that exposure to 5–500 g/mL MWCNTs improved the growth of tobacco plant cells by approximately 55–60% as compared with that in the control group. Through physiological and morphological assays, the positive effects of MWCNTs were assessed in various crops, including red spinach, lettuce, rice, cucumber, chili, soybean, and lady’s finger, in hydroponic culture. As illustrated in Fig. 3C, the root and shoot lengths of red spinach and lettuce were drastically reduced after 15 days of hydroponic cultivation with exposure to 1000 mg/L and 2000 mg/L MWCNTs. The most sensitive crops to MWCNTs were red spinach and lettuce, followed by rice and cucumber; the MWCNTs were shown to have marginal or no toxicity to chili, lady’s finger, and soybean [146]. Another study showed that supplementation of CNTs in soil increased the flower number and fruits by two-fold, whereas the size and quality of leaves were not changed compared with those grown in control soil (Fig. 3F) [53].

The effects of four different carbon NMs, including activated carbon, SWCNTs, MWCNTs, and graphene oxide (GO), on nodulation and nitrogenase activity in a rhizobium-legume system illustrated in Fig. 3G. The results demonstrated that under non-symbiotic conditions, SWCNTs and GO inhibited bacterial growth and the development of plant roots, whereas MWCNTs (50, 100, 150, 200, 500 µg/mL) enhanced stem and nodule development by activation of nitrogenase activity in the rhizobium-plants interaction [147]. The researchers hypothesized that CNT surface charges may stimulate the production of water channel proteins in tomato plants, thus improving the plant’s water transport and absorption [53, 148, 149]. Zhai et al. [150] investigated the effect of three types of MWCNTs (p-MWCNTs, positively charged NH_2_-MWCNTs, and negatively charged COOH-MWCNTs), which were directly uptaken and translocated to the roots, stems, and leaves of maize and soybean at concentrations of 10–50 mg/L during 18 days of exposure in hydroponic conditions. Transmission electron microscopy (TEM) revealed that the MWCNTs aggregated in xylem and phloem cells, as well as within intracellular regions (cytoplasm, cell wall, cell membrane, chloroplast, and mitochondria). Further, MWCNTs enhanced maize growth but inhibited soybean growth. Water transpiration was nearly twice as high in maize subjected to 50 mg/L MWCNT-COOHs as it was in the control maize [150]. Furthermore, pre-germinated wheat seeds were soaked with an MWCNT solution at 10–160 µg/L for 4 h, resulting in faster root growth and better vegetation [151]. Rice seeds were treated with varied concentrations of MWCNTs (70, 80, and 90 g/mL) and were primed with oxidized MWCNTs having a diameter of 14–30 nm and a length of 200–300 nm. The plants treated with MWCNTs had denser stomata and longer roots, which resulted in better growth and rapid water and mineral intake, thus increasing crop output. The content of chlorophyll and photosynthetic activity also increased as a result of the increase in the number of vascular tissues [152]. Rahmani et al. [153] further discovered that MWCNTs (50 mg/L) significantly improved photosynthetic pigments and activated vital enzymes in *Salvia verticillata*.

SWCNTs (10–40 mg/L) were reported to alter the seed germination rate in salvia (*Salvia macrosiphon*), pepper (*Capsicum annuum*), and tall fescue (*Festuca arundinacea*), which was likely caused by seed coat perforation [132]. When compared to the effects of SWCNTs or chloroplast alone, SWCNT-chloroplast assemblies boosted the rate of electron transport in the leaves and promoted higher photosynthetic activity. The effect of SWCNTs on germination rates in various plant species differed with exposure to different concentrations of SWCNTs. SWCNTs at 10 mg/L and 30 mg/L promoted the germination of pepper and fescue, respectively, but had no effect on maize (*Zea mays*) seed germination at a concentration of 20 mg/L. SWCNTs were also shown to accelerate the growth of the seminal roots in maize plants by promoting gene expression, and SWCNT treatment caused upregulation of the expression of epigenetic modification enzyme genes in a dynamic and selective manner, which is similar to the response of plants to other stresses [154]. Similarly, SWCNTs improved the chlorophyll content and photosynthetic rate, resulting in enhancement of rice growth at a minimal concentration of 20 mg/L [155]. Table 1 summarizes the studies that have focused on CNT applications in seed germination and the growth of plants.

**Table 1:** Studies examining the effects of CNTs on plant growth and development

CNT type	Concentration	Plant species	Growth conditions	Effects	References
CNT	10–40 µg/mL	Tomato	MS media	Enhanced seed germination and plant growth	[130]
CNT	0–40 µg/mL	Tomato, onion, radish, turnip	Laboratory and greenhouse	Improved seed germination and seedling growth	[60]
CNT	0.0–0.1 µg/mL	Date palm	In vitro	Increased shoot length and leaf number	[251]
SWCNT	Varied concentrations	Carrot, cabbage lettuce, onion, tomato, cucumber	In vitro	Enhanced the roots/shoot length, increased growth	[63]
SWCNT	10–40 µg/mL	Salvia, pepper, tall fescue	laboratory and greenhouse	Increased the seed germination rate	[132]
SWCNT	0–50 µg/mL	Tomato	In vitro	Increased, chlorophyll content, weight, and height of roots/shoots	[144]
SWCNT	5 and 20 µg/mL	Rice	In vitro	Seedling development	[155]
SWCNT	20, 50 ,100 µg/mL	Maize	In vitro	Enhanced growth of seminal roots	[154]
MWCNT	0–1000 µg/mL	Red spinach	Hydroponic culture	No effect on plant growth	[235]
MWCNT	0–2000 µg/mL	Red spinach, lettuce, rice, cucumber, chili, lady finger, soybean	Hydroponic culture	Enhanced root or shoot length, decreased plant growth	[146]
MWCNT	5–500 µg/mL	Tobacco	MS media	Improved growth and plant dry biomass	[131]
MWCNT	2.3 × 10^− 3^ mg/mL 46 × 10^− 3^ mg/mL	Mustard	In vitro	Enhanced root size, increased plant growth	[139]
MWCNT	0, 500, 1000, or 5000 mg/kg	Corn	Soil	Increased plant growth and plant dry biomass	[57]
MWCNT	25, 50,100 µg/mL	Barley, corn, rice, soybean, switchgrass, tomato	MS media	Activation of early seed germination	[140]
MWCNT	50, 100, and 200 µg/mL	Barley, soybean, corn	MS media	Activation of early seed germination, increased root length and biomass	[148]
MWCNT	10–160 µg/mL	Wheat	In vitro	Increased root length of wheat seedlings	[151]
MWCNT	40 − 2560 mg/L	Alfalfa, wheat	MS media	Increased root length and plant growth	[58]
MWCNT	20 and 50 µg/mL	Wheat, maize peanut, and garlic	Soil	Enhanced root/shoot elongation	[252]
MWCNT	70, 80 and 90 µg/mL	Rice	Laboratory and field	Increased root length, chlorophyll content, and photosynthetic activity	[152]

*CNT* carbon nanotube; *SWCNT* single-walled carbon nanotube; *MWCNT* multi-walled carbon nanotube

## CNTs as antimicrobial agents against pathogens

**Fig. 4:**
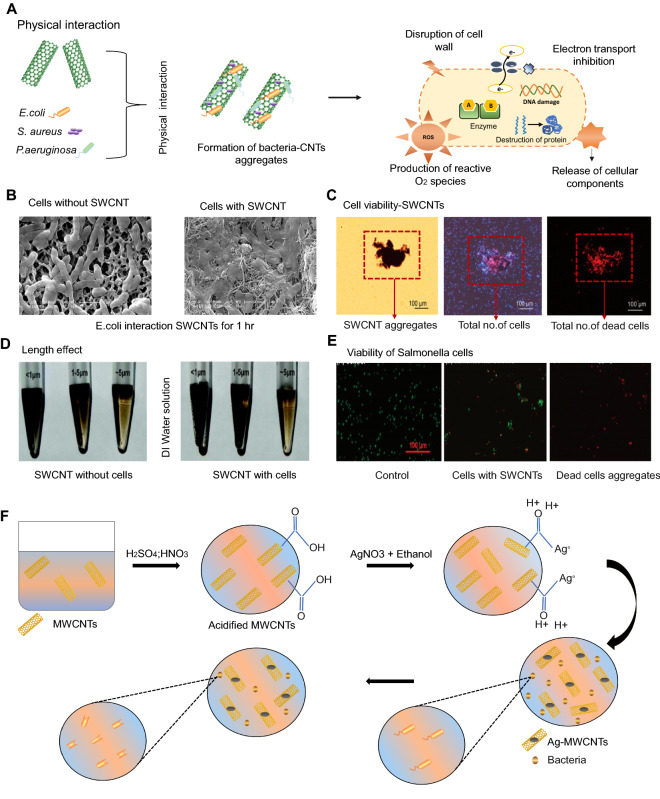
Systematic illustration of the antimicrobial activity of carbon nanotubes (CNTs) against various pathogens. **A** Physical interaction of CNTs with microbes leads to membrane damage and the release of biological components. **B** Images of *Escherichia coli* cells treated without (left) and with (right) single-walled CNTs (SWCNTs) for 60 min captured using scanning electron microscopy (SEM). Scale bar = 2 μm [162]. **c** Measurement of cell viability after exposure to SWCNTs (5 g/mL) in a 0.9% NaCl isotonic solution. Image of SWCNT aggregates at the microscopic level from a total cell fluorescence microscope (cells were stained with propidium iodide and DAPI). Using the fluorescence microscope, a fluorescence image of dead cells was captured (stained with propidium iodide only) [162]. **D** SWCNTs of < 1 μm, 1–5 μm, and ∼5 μm in a deionized (DI) water solution at a concentration of 100 µg/mL. *Salmonella* cells (6.0 × 10^8^ colony-forming units/mL) were combined with three different lengths of SWCNTs (100 µg/mL) in DI water for 20 min [161]. **E** Images of *Salmonella* cells stained with the Live/Dead bacterial viability kit without and with SWCNTs: cells without SWCNTs in the control sample, live (green) and dead (red) cells in the sample of cells with SWCNTs of 1–5 μm [161]. **F** The synthesis of silver (Ag)–multi-walled CNTs (MWCNTs) and evaluation of their antibacterial activity [180]

Different infectious plant diseases are mainly caused by pathogenic organisms such as viruses, bacteria, fungi, nematodes, as well as insects and parasitic plants. These infectious plant diseases are constantly responsible for severe damage to plant growth and yield losses in major crops including cereals, vegetables, and industrial crops [156, 157]. For instance, *Alternaria solani* is one of the most devasting disease causes stem and fruit rot as well as leaf blight, which affect plant growth [158]. In general, the antibacterial action of nanoparticles is largely determined by their composition, surface functionalization, inherent properties, and microbe type [159, 160]. Various studies have demonstrated that different sizes and diameters of CNTs have dramatically variable antibacterial efficiency [161–163]. The antibacterial effectiveness of CNTs is further influenced by extrinsic parameters such as the CNT dispersion ability, culture medium, bacterial type, CNT dosage, reaction time, and the mode of action between bacteria and CNTs [164–166]. CNTs are being extensively studied as potential antibacterial agents owing to their stability and efficient biological characteristics [167, 168]. CNTs show significant antibacterial activity by causing physical and chemical harm to bacteria. Direct contact causes physical damage to the membrane as well as changes in cell shape, resulting in cytoplasmic leakage, enzyme and electrolyte release, and lipid breakdown, all of which lead to the microorganism’s cell death, [169–171] as demonstrated in Fig. 4A.

According to several studies, CNTs possess significant antibacterial activity. Kang et al. [164] present first evidence that highly purified, pristine SWCNTs have significant antibacterial properties. The results revealed that cell membrane damage caused by direct contact with SWCNT aggregates is the most likely cause of bacterial cell death by employing a pristine SWCNT with a narrow diameter distribution. This discovery could be useful in the development of antibacterial materials using SWNTs as building blocks. CNTs could be an alternative for the control of pathogens since they have strong antibacterial action and induce the activation of the antioxidant defense system in plants. For instance, the incidence and severity of *A. solani* on tomato crop were studied using MWCNTs. Results revealed that MWCNTs improved the antioxidant defense mechanism as a result of increased in content of ascorbic acid, flavonoids, and the glutathione peroxidase enzyme. The net photosynthetic capacity and water use efficiency also enhanced by the utilization of MWCNTs [172]. Wang et al. [173] investigated the antimicrobial activity of CBNs [SWCNTs, GO, reduced GO, and fullerene (C_60_)] against a copper-resistant plant pathogenic bacterium (*Ralstonia solanacearum*). The antibacterial activity of the SWCNT dispersion was found to be the strongest, followed by that of GO, MWCNTs, reduced GO, and C_60_. According to the antibacterial mechanism of SWCNTs and GO, damage to the cell membrane causes the release of cytoplasmic materials from the bacterium, which is the main reason for the inactivation of *R. solanacearum* bacterial cells.

Using confocal microscopy, flow cytometry, and antibiotic tolerance experiments, it was found that sub-lethal dosages (2 mg/L) of MWCNTs increased the aggregation of *Pseudomonas aeruginosa* into multicellular clusters that can cause diseases in plants and animals including humans. By contrast, the antibiotic tolerance of these “young” bacterial-CNT aggregates was comparable to that of CNT-free cultures [174]. Another study demonstrated that the size (diameter) of CNTs is a major factor determining their antibacterial properties, and cell membrane damage caused by direct interaction with CNTs is the most likely CNT cytotoxicity mechanism. According to experiments with well-characterized SWCNTs and MWCNTs, SWCNTs are substantially more hazardous to bacteria than MWCNTs. In the presence of both MWCNTs and SWCNTs, *Escherichia coli* expresses huge amounts of stress-related proteins, with the quantity and degree of expression being much higher in the presence of SWCNTs. SWCNTs’ increased bacterial toxicity may be due to (1) a smaller nanotube diameter that allows for partial penetration of nanotubes into the cell wall, (2) a larger surface area for contact and interaction with the cell surface, and/or (3) unique chemical and electronic properties that convey greater chemical reactivity (Fig. 4B, C) [162]. The antibacterial activity of CNTs depends on the bacterial species. The differential insensitivity of microorganisms to CNT exposure could be due to differences in cell wall thickness and cell membrane constituents [160]. Physical and chemical changes in MWCNTs by dry oxidation and acid treatment were shown to change their dispersion capacity and increase their antibacterial activity [175].

The effect of the length of CNTs on antibacterial activity has been investigated in several studies. Yang et al. [161] investigated the antimicrobial activity of three different lengths (< 1 μm, 1−5 μm, and ∼ 5 μm) of SWCNTs. Results showed that a longer SWCNT has greater aggregation and antibacterial capabilities at the same weight concentration. The fluorescence and SEM images revealed that SWCNTs with longer length aggregated with bacterial cells more effectively, whereas short-length SWCNTs tended to aggregate themselves without involving many bacterial cells. Furthermore, the antibacterial activity of longer SWCNTs was more dependent upon concentration and treatment time (Fig. 4D, E). However, on solid surfaces, shorter CNTs (1–5 μm) exhibited stronger antibacterial activity than longer CNTs. CNTs of shorter lengths also improved interactions with microbes and caused degradation of the cell wall [176].

Oxidative stress is one of the major chemical effects caused by CNTs, which is due to ROS generation. Since the NM interacts with microbes, oxidative stress is produced, resulting in cell death [177]. The surface charge of CNTs has been shown to promote their antibacterial properties, which is based on their ability to disrupt the integrity of the cell membrane [178]. Bing et al. [179] found that the surface charge of CNTs affected both bacterial mortality and antimicrobial activity. In fact, after the contact between charged dots and bacterial cells, the production of ROS such as hydroxyl radicals was determined to be the key factor contributing to the inhibition of bacterial growth [179]. Moreover, MWCNTs were treated with silver (Ag) particles and a mixture of acids through the chemical reduction of Ag cations by an ethanol solution, and the antibacterial activity of these Ag-MWCNTs (30 µg/mL) was examined against bacterial species (*Methylobacterium* spp. *and Sphingomonas* spp.), showing that a low concentration of Ag-MWCNTs could effectively hinder bacterial growth (Fig. 4F) [180]. Another study investigated the protective role of carbon-based NPs (C_60_ and CNTs) against single-stranded RNA *Tobacco mosaic virus* (TMV) at concentration of 100, 200, and 500 mg L^−1^ for a 21-day foliar exposure. Plants treated with CNTs and C_60_ (200 mg L^− 1^) exhibited normal phenotype, prevented the reproduction of TMV, and limited its spread to apical tissues in *Nicotiana benthamiana.* Fluorescence measurement of CNTs and C_60_ (200 mg L^− 1^) treated plants indicated photosynthesis equivalent to healthy controls. CNTs and C_60_ caused a 33–52% increase in the defense-related phytohormones abscisic acid and salicylic acid, as well as a 94–104% increase in the transcription of genes involved in phytohormone production in treated plants [181]. A detailed summary of the applications of CNTs as antimicrobial agents is provided in Table 2.

**Table 2:** CNTs as antimicrobial agents, along with their mechanisms and characteristics

CNT types	Species	Concentration	Action mechanism	Antimicrobial efficiency (%)	References
CNT	TMV	200 mg/L	Damages optical tissues and reduces the reproduction of TMV	–	[181]
MWCNT	*Alternaria solani*	100 mg/L	Enzymatic degradation	–	[172]
MWCNT	TuMV	200 mg/L	Inhibits viral proliferation, decreases the TuMV protein coat	15–60	[253]
MWCNT	*Escherichia coli* *Pseudomonas aeruginosa* *Bacillus subtilis*	100 µg/mL	Membrane integrity lost due to piercing/trapping	–	[254]
MWCNT	*Pseudomonas fluorescens*	–	Inhibits bacterial adhesion under electrochemical potential	44	[255]
SWCNT	*Salmonella typhimurium*	100 µg/mL	Rupture of outer membrane	–	[161]
SWCNT	*Ralstonia solanacearum*	1 mg/mL	Damages the cell membrane causing the release of cytoplasmic contents	–	[173]
SWCNT	*E. coli*	0.1–0.2 mg/mL	Cell membrane damage due to formation of CNT aggregates	–	[256]
SWCNT	*E. coli*	5 µg/mL	Reduction in cellular integrity	80.1	[162]
SWCNT	*Bacillus anthracis*	200 µg/mL	Disruption of cell membrane	81.2	[162]
SWCNT	*Staphylococcus epidermidis*	1/70 CNT/polymer	Cells are deactivated due to loss of cell viability	98	[257]
SWCNT	*Salmonella typhimurium*, *B. subtilis*, *Staphylococcus aureus*	200–250 µg/mL	Formation of needle-like aggregates around the cell as result cell become damage	~ 7 log	[165]
SWCNT	*Salmonella typhimurium*	62.5 µg/mL	Inhibition of some genes responsible for metabolism and outer membrane integrity	–	[258]

*CNT* carbon nanotube; *MWCNT* multi-walled carbon nanotube; *SWCNT* single-walled carbon nanotube; *TMV* *tobacco mosaic virus*; *TuMV Turnip mosaic virus*; *E. coli Escherichia coli*

## CNTs for genetic material or drug delivery

**Fig. 5:**
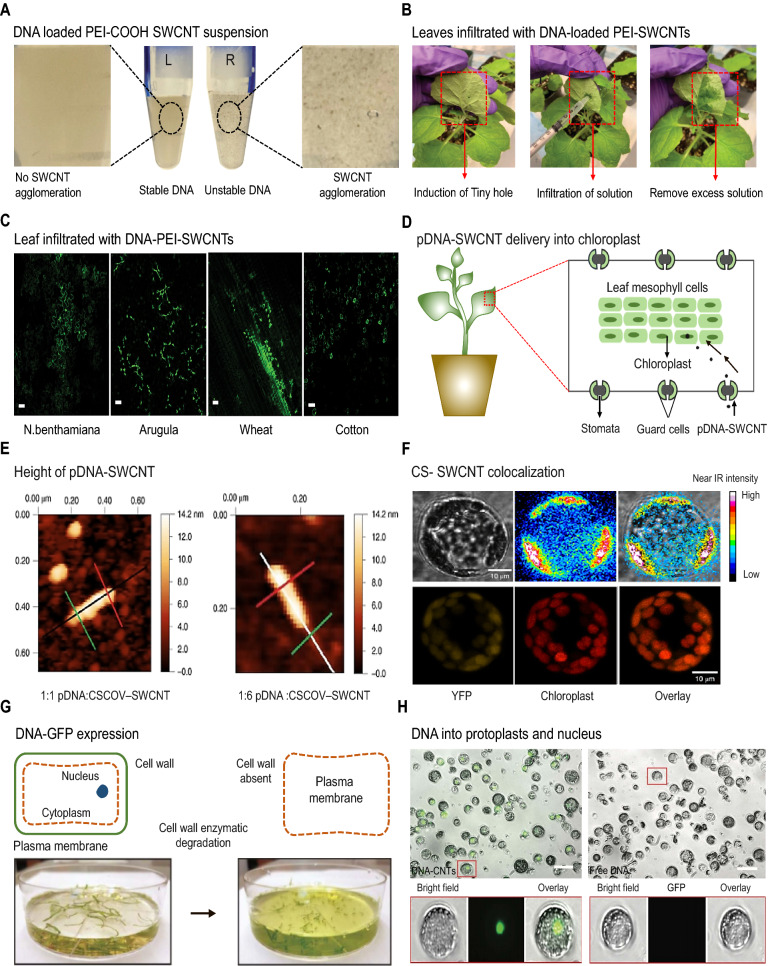
Carbon nanotubes (CNTs) serve as carriers for genetic material or drug delivery. **A** The image on the left illustrates the stability of DNA loading on polyethyleneimine (PEI)-single-walled carbon nanotubes (SWCNTs), whereas the image on the right represents the instability of DNA loading on PEI-SWCNTs, with significant SWCNT agglomeration [186]. **B** Infiltration of leaves with DNA-loaded PEI-SWCNTs. By infiltrating a higher volume of DNA-PEI-SWCNT solution, the area of penetration can be enhanced [186]. **C** Confocal microscopy images of wild-type *Nicotiana benthamiana* (Nb), arugula, wheat, and cotton leaves infiltrated with DNA-PEI-SWCNTs to measure green fluorescent protein (GFP) expression levels in the leaf lamina of each plant species. Scale bars = 50 μm [185]. **D** Stomata pores allow plasmid DNA (pDNA)–SWCNT complexes to enter the leaf mesophyll. Electrostatic interactions help to condense negatively charged pDNA on the positively charged surface of chitosan-complexed SWCNTs [45]. **E** Atomic force microscopy (AFM) image of a 1:1 pDNA:CSCOV–SWCNT complex with representative height; 1:6 pDNA:CSCOV–SWNT AFM height image standard deviations (n = 3) are represented by the error bars [45]. **F** Fluorescence confocal micrographs of isolated protoplasts demonstrate yellow fluorescent protein (YFP) expression from pDNA coupled to CSCOV–SWCNTs (1:6 pDNA:SWCNT w/w ratio) after 24 h [45]. **G** CNT-mediated DNA delivery into isolated protoplasts and subsequent GFP expression. Enzymatic cell wall degradation extracts of intact and healthy protoplasts from arugula leaves [193]. **H** Protoplasts incubated with DNA-CNTs display robust GFP expression in the nuclei, whereas protoplasts cultured with free pDNA without CNT nanocarriers do not show strong GFP expression. Red boxes represent areas of interest that are highlighted and expressed with bright-field, GFP, and overlay channels. Scale bars = 25 μm [193]

CNTs have substantial potential as a nanocarrier or delivery vehicle for genetic material or nutrients into plant cell organelles such as the vacuole and plastids due to their low toxicity and their capacity to cross the plant cell wall, cell membrane, and cell organelles [182, 183]. The most commonly used technique for genetic engineering in plants is *Agrobacterium*-mediated gene delivery; however, *Agrobacterium* does not infect numerous crops [184]. Another study compared the transformation efficiency between *Agrobacterium* and CNTs, showing that CNTs up to 20 nm can pass through plant cell walls at least in one dimension, indicating their potential as a tool for gene transformation in plants [185]. Nanocarrier-based nutrient delivery is a promising technique for plant improvement. For instance, Liu et al. [43] first used SWCNTs as plant gene delivery vehicles in 2009. Confocal fluorescence images revealed that cellular uptake of both SWCNT/FITC and SWCNT/single-stranded DNA (ssDNA)-FITC conjugates were taken up by *Nicotiana tabacum* cells, implying that SWCNTs can infiltrate the integral plant cell walls and cell membranes without the need for external assistance such as a gene gun. Moreover, the results suggested that SWCNTs could deliver different cargoes into plant cell organelles [43]. With this ability, CNTs are now being suggested for use as nutrient delivery in a slow and controlled manner to reduce the loss of excess nutrients and enhance plant growth under stress conditions [52]. Functionalized high-aspect-ratio CNT nanoparticles demonstrated efficient plasmid DNA delivery into intact plants of several species, including arugula, wheat, and cotton, resulting in high protein expression levels in a variety of non-model plant species and providing a protocol to deliver plasmid DNA in a species-dependent manner (Fig. 5A, B) [186]. Pristine and chemically functionalized high-aspect-ratio NMs were used to demonstrate efficient diffusion-based biomolecule transport into whole plants of numerous species. Leaves and protoplasts from *Nicotiana benthamiana*, *Eruca sativa* (arugula), *Triticum aestivum* (wheat), and *Gossypium hirsutum* (cotton) showed efficient DNA delivery and high protein expression without transgenic integration (Fig. 5C). They also showed that NMs not only help biomolecules enter plant cells but also protect polynucleotides from being degraded by nucleases [185].

Giraldo et al. [32] examined the feasibility of ssDNA-loaded SWCNTs to penetrate *Arabidopsis thaliana* leaves. They injected the complex using a syringe into the surface of leaves and discovered that CNTs of a specific size and charge could penetrate membranes without the use of a gene gun. Insertion of SWCNTs resulted in a three-fold increase in the photosynthetic rate of *Arabidopsis* leaves compared with that of control leaves. This effect was mainly due to the presence of the SWCNTs inside the lipid envelopes of the chloroplast, which increased the rate of maximum electron transport and enhanced the capabilities of photoabsorption. Likewise, it was examined whether immobilized cellulose on CNTs could be incubated with plant cells. Nanoholes were developed on the plant cell wall, which facilitated the delivery of biomolecules across the cell wall [187]. For successful DNA transport in plant cells, an optimized amount of non-covalently functionalized SWCNTs and MWCNTs is important [188]. Yellow fluorescent protein signals indicated that the SWCNTs were able to successfully permeate plant cell walls and transport the reporter gene inside cells from a variety of plant tissues, whereas the MWCNTs could only enter protoplast cell membranes.

By using a lipid exchange envelope and penetration model, NMs were modified for the selective delivery of a biological material to certain plant organelles via passive delivery. The researchers [45] synthesized a variety of chitosan-complexed SWCNTs (CS-SWCNTs) by non-covalently wrapping or functionalizing the SWCNTs via covalent binding to chitosan molecules, which enhanced the efficiency of loading and transport of DNA to the chloroplast (Fig. 5D–F). Through electrostatic interactions, chitosan binds to plasmid DNA and protects it from nuclease activity. The moderate pH of the cytosol facilitated the binding of plasmid DNA to CS-SWCNTs, whereas an acidic pH and weaker electrostatic interactions in the chloroplast caused the cargo to be released from the SWCNTs [45]. Another study examined the ability of synthetic chimeric peptides and arginine-functionalized SWCNTs (Arg-SWCNTs) to deliver DNA into functional tobacco root cells. Owing to their nano-cylindrical structure, Arg-SWCNTs and the plasmid DNA adsorbed onto the cell surface and could cross the barriers of the plant cell. Observations of green fluorescent protein (GFP) expression and western blot analysis demonstrated that Arg-SWCNT–mediated DNA can be transported in tobacco root cells [189]. In 2016, Ochoa-Olmos et al. [190] attempted to transport DNA to *Nicotiana tabacum* protoplasts and the cell wall using SWCNTs and MWCNTs; however, the SWCNTs changed the protoplasts as well as the cell walls, and the MWCNTs were less effective in gene transfer.

CNTs can interact with a variety of cell types and can be taken up through endocytosis. MWCNTs were shown to influence the accumulation of contaminants by acting as contaminant carriers in crops [191]. Furthermore, the compounds that were adsorbed by the plants via the MWCNTs could be released within the plant to effectively provide routes for the delivery of genetic material or drugs to specific sites of intact plants [191]. MWCNTs were also shown to influence the transcription and translation of particular genes and consequent phenotypes [192]. Demirer et al. [193] revealed that a CNT-based plasmid DNA delivery platform allows for the quick and passive delivery of DNA into protoplasts as well as transgenic expression, with high efficiency and no detrimental impacts on protoplast viability. For this procedure, intact and healthy protoplasts were isolated from arugula leaves (Fig. 5G), and the extracted protoplasts were then treated for 24 h with plasmid DNA-CNTs generated by dialysis. Fluorescence microscopy showed that GFP was strongly expressed in the nuclei of protoplasts treated with DNA-CNTs (Fig. 5H), but was not detected in protoplasts cultured with free plasmid DNA [193]. A recent study demonstrated that CNTs could also be utilized for gene transfer in German chamomile cells, where the surface of the CNTs was modified in cationic form using polyethyleneimine, and the ssDNA-FITC conjugate bound to the CNT surface via electrostatic interactions. The application of ultrasound aided the nucleic acid-coated CNTs to significantly increase the transfer efficiency of ssDNA-FITC because the ultrasound waves enhanced the gene transfer rate by producing a cavity at the nanoparticle surface. Furthermore, gene transfer efficiency was improved by cationic nanoparticles as they could protect the DNA against ultrasound waves as compared with the effect of using ultrasonic waves alone [194]. A detailed summary of the applications of CNTs in gene delivery to plants is provided in Table 3.

**Table 3:** Summary of various CNT-based gene delivery studies in plants

CNT type	Plant species	Cargo	Target organ	Delivery method	References
CNTs	*N. tabacum*	DNA	Protoplast and cell wall	Passive	[190]
CNTs	Arugula, wheat, cotton	Plasmid	Leaves	Passive	[186]
CNTs	*N. tabacum*	YFP plasmid	Protoplast, leaf	Passive	[188]
CNTs	*N. tabacum*, *Eruca sativa*, *Triticum aestivum*, and *Gossypium hirsutum*	GFP, Cy3 DNA	Protoplast, leaf	Infiltration with syringe	[185]
CNTs	*E. sativa*, *Nasturtium officinale*, *N. tabacum*, *Spinacia oleracea*	Plasmid	Mesophyll protoplast	Passive	[45]
CNTs	German chamomile	ssDNA-FITC	Chamomile cells	Ultrasound	[194]
SWCNT	*Arabidopsis thaliana*	ssAT-SWCNT	Chloroplast	Passive	[32]
SWCNT	*N. tobacum*	GFP plasmid	Root	Passive	[189]
SWCNT	*N. tabacum*	SWCNT/FITC	Cell wall and membrane	Fluidic-phase endocytosis	[43]
SWCNT	*N. benthamiana*	ssRNA	Leaf	Passive	[259]

*CNT* carbon nanotube; *YFP* yellow fluorescent protein; *GFP* green fluorescent protein; *ssDNA* single-stranded DNA; *FITC* fluorescein isothiocyanate; *ssAT* single-stranded *A. thaliana*; *SWCNT* single-walled carbon nanotube; *ssRNA* single-stranded RNA; *N. tabacum* *Nicotiana tabacum*; *N. benthamiana Nicotiana benthamiana*

## CNTs as nanosensors

CNT-based biosensors have significant benefits over commercially available sensors such as metal oxides, silicon, and others, including a large surface area ratio, outstanding luminescence qualities, fast reaction time, and high stability [195]. Plant phenotype analyses have focused on plant morphological, functional, or physiological factors to find features that increase crop tolerance to external stress and disease; however, there are less techniques available for monitoring plants’ internal chemical signals linked with stress [196, 197]. CNT-based nanosensors have been used in agriculture to detect soil humidity, pesticide residues, proteins or hazardous materials, and for pest identification [198–200]. The properties of CNTs based on dimension promote the ultrasensitive detection of analytes because all atoms are surface atoms, and minor changes in chemical composition can radically modify the optical and electrical characteristics [201]. Nanosensors are more useful for smart and sustainable agriculture because they have a low detection limit and high sensitivity. SWCNTs have previously been shown to be promising instruments for biosensing applications based on chirality-dependent fluorescence in the near-infrared region (NIR) [201–203]. The surface chemistry of SWCNTs could be altered by chemical functionalization of peptides, lipids, nucleic acids, and proteins [204, 205].

**Fig. 6:**
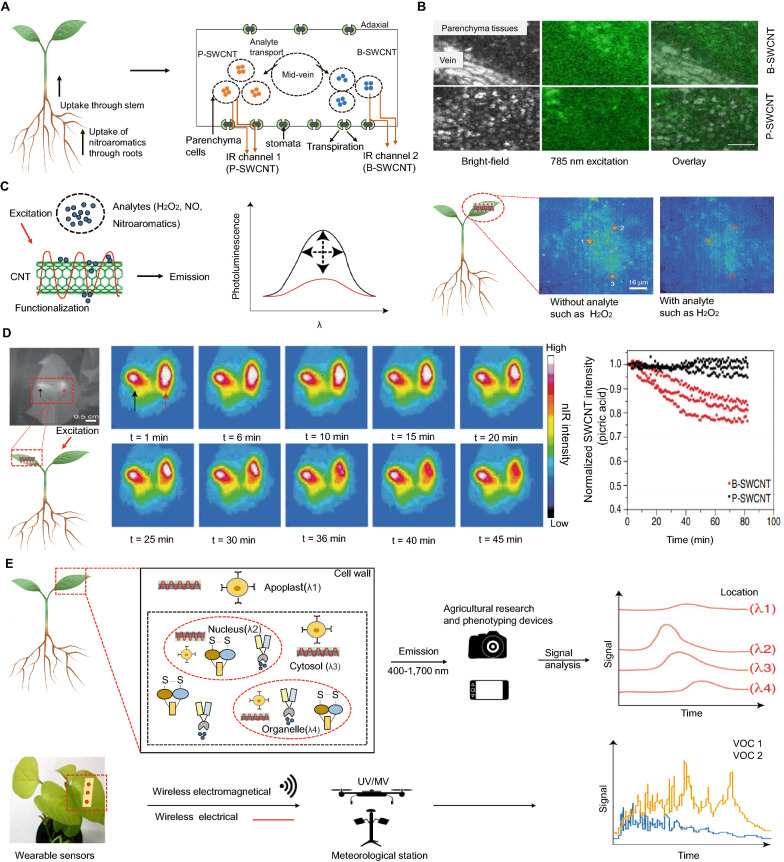
Applications of carbon nanotubes (CNTs) as biosensors. **A** The plant serves as a fluidic device and an environmental sampler. Water and other analytes are carried by the roots into the stem and toward the leaf tissues via the plant vasculature when the leaves transpire. Bombolitin II-modified single-walled carbon nanotubes (B-SWCNTs), serving as an active sensor, and polyvinyl alcohol-modified SWNCTs (P-SWCNTs), serving as a reference sensor, infiltrate the leaves via the abaxial surface on each side of the leaf midrib [206]. **B** After infiltration, SWCNTs are found within the leaf parenchyma tissues, as evidenced by the fluorescence detected when the leaf was excited at 785 nm. Scale bar = 0.2 mm [206]. **C** Because of the nanomaterial carbon lattice, analyte interaction with SWCNT sensors generates fluctuations in near-infrared (NIR) fluorescence intensity or wavelength shifts. H_2_O_2_ monitoring in vivo was accomplished using SWCNT NIR fluorescence intensity fluctuations in *Arabidopsis* leaf slices with high spatial (> 0.5 m) and temporal (> 0.5 s) resolution [208]. **D** Plant leaves embedded with SWCNTs act as nitroaromatic detectors, such as for picric acid. Internal controls consist of P-SWCNTs (black arrows), whereas B-SWCNTs (red arrows) monitor picric acid in real time with high spatiotemporal resolution [206]. **E** Plant subcellular sensors based on smart nanobiotechnology can monitor plant chemical signaling using phenotyping technology, which could aid in the selection of desirable plant features for high yield and stress tolerance [211]

Another study demonstrated that SWCNTs coated in polyvinyl alcohol (PVA) and Bombolitin II infiltrated the leaf lamina of 3-week-old spinach plants to turn them into nitroaromatic detectors (hereafter referred to as Bombolitin). PVA–SWCNTs (P-SWCNTs) and Bombolitin–SWCNT (B-SWCNTs) infiltrated two sections of a single leaf’s lamina separated by the mid-vein using a needleless syringe, where they became embedded into the parenchyma tissues of the leaf lamina [32]. The NIR fluorescent signal of P-SWCNTs is picric acid-invariant, allowing its emission to serve as a plant reference signal. In reaction to picric acid, the NIR fluorescence intensity of B-SWCNTs decreases, allowing it to function as an active sensor. As contaminant nitroaromatics travel up the stem via the roots and into the plant vasculature, they finally reach the leaf tissues and come into contact with the embedded sensors (Fig. 6A, B, D). The presence of picric acid is indicated by a decrease in the intensity of B-SWCNT fluorescence, which is detected by a detector. Before developing a standoff detection set-up for the far-field monitoring of nitroaromatics, the plant’s ability to operate as a groundwater sampler of picric acid was first established using NIR microscopy [206].

H_2_O_2_ is a typical ROS molecule that may easily permeate membranes via water channels [207]. An NIR fluorescence sensor based on SWCNTs was constructed to detect H_2_O_2_ in *Arabidopsis thaliana* leaves. The sensor’s NIR fluorescence response was quenched by H_2_O_2_, and no other stress-related signaling chemicals elicited the same reaction. This H_2_O_2_ sensor enabled the *in vivo* and remote NIR imaging of plant conditions in response to different stimuli such as a pathogen-related peptide, high light, and ultraviolet-B light, but was not able to detect leaf injury (Fig. 6C) [208]. Through real-time measurements of single stomatal opening dynamics, microfluidic printed SWCNT ink on the leaf epidermis may detect the plant water status and the start of drought stress. This wearable sensor is composed of two printed contact pads and a stripe that spans a single stoma, and is sensitive to minor changes in stomatal opening and closing latency during drought [209]. Wearable SWCNT-graphite sensors can be operated by radiofrequency in response to gas molecule concentrations as low as 5 ppm, enabling wireless monitoring with electronic devices with no power consumption [210]. Chemoresistive sensors built on SWCNTs and outfitted with copper complexes are reversible, enabling the long-term monitoring of sub-parts per million quantities of ethylene, a plant hormone that serves as a major indicator of fruit ripening. Plant volatile organic chemical-sensing devices based on CNTs, such as ethylene, are now commercially accessible for agriculture applications. However, they have not yet been interfaced directly with crops for monitoring plant signaling chemicals (Fig. 6E) [211]. Dong et al. [212] proposed an electrochemical sensor based on MWCNTs–CeO_2_–Au for both the enrichment and detection of methyl parathion at ultra-trace levels in soil and water. The excellent conductivity, increased effective surface area, and catalytic activity enabled methyl parathion enrichment and very sensitive electrochemical stripping detection of approximately 3.02 × 10^− 11^ M under optimal conditions, suggesting the high sensitivity of the synthesized sensor [212].

## CNT uptake and defense mechanism against environmental stresses

In numerous plant species, applying nanoparticles at pre-optimized rates promotes seed germination, growth, and yield production. The alteration of stress-tolerant genes and stress proteins by nanoparticles contributes to tolerance to various biotic and abiotic stresses in plants [32, 213]. In this section, we discuss the mechanisms of CNT uptake and translocation, as well as defense mechanisms against various environmental stresses in crops.

### Uptake and translocation of CNTs in crops

**Fig. 7:**
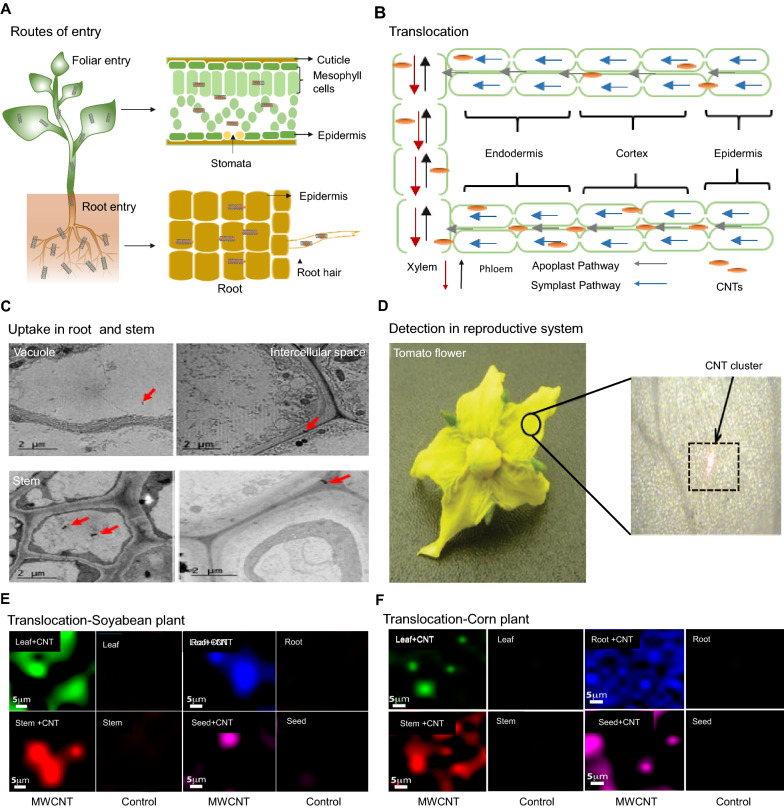
Schematic representation of the uptake and translocation mechanisms of carbon nanotubes (CNTs) via different routes in crops. **A** Different routes of entry of CNTs into crops, including foliar and root entrance. **B** CNT movement in several organs of plant leaves and roots. **C** Transmission electron microscopy characterization of multi-walled carbon nanotube (MWCNT) uptake in broccoli plants cultivated for 7 days with MWCNTs (10 mg/L) in nutrient solution. Arrows point to MWCNTs in the intercellular space, vacuole, and cytoplasm of the roots and stems [54]. **D** Spectroscopic characteristics of individual CNTs to detect CNT aggregates in tomato flowers using Raman scattering [53]. **E** Raman spectroscopy to identify MWCNT translocation inside soybean plants. Slices of various plant tissues, including the leaves, stems, roots, and seeds, were prepared and studied using Raman spectroscopy and point-by-point mapping of selected locations [229]. **F** Raman spectroscopy to identify MWCNT translocation inside maize plants. Slices of various plant tissues, including the tassels, leaves, stems, roots, and seeds, were prepared and studied with Raman spectroscopy using point-by-point mapping of selected locations [229]

NM absorption and distribution in plants is a growing concept of research interest. Foreign compounds can be protected by the plant cell wall, which is made up of a network of cellulose fibrils. As nanoparticles or nanoparticle aggregates have a smaller diameter than the cell wall’s pore diameter, they can pass through the cell via the apoplast route [49, 214]. Plant species, age, growth conditions, physicochemical quality, functionalization, stability, and distribution of nanoparticles all influence their uptake, translocation, and accumulation [215, 216]. A schematic of the uptake of CNTs and their translocation through different routes in crops is shown in Fig. 7A, B.

A recent study demonstrated that MWCNTs can alter the expression of genes that play vital roles in water transport and stress signaling [217]. MWCNTs can activate the gene encoding the water channel protein aquaporin, which is considered one of the most important membrane proteins that facilitate the transport of water in plants [218]. MWCNTs can stimulate the growth of tomato and wheat seedlings by producing more new pores in the cell wall and plasma membrane, which are responsible for water transport [219, 220]. Another study comprehensively investigated the impact of MWCNTs on the accumulation and transport of variety of organic contaminants from the soil to various parts of the mustard plant, such as organochlorine pesticides, organophosphorus pesticides, pyrethroid insecticides, medicines, and personal care items. Mustard plants were irrigated with different concentrations (1 and 10 µg/mL) of MWCNTs suspended in aqueous solution to test the accumulation rate and relative kinetic process in living plants leaves. The accumulation kinetics and individual concentrations of the contaminants in leaves after 16 days of exposure showed that bioaccumulations of most contaminants in the leaves increased by 10–30% (1 µg/mL) and 20–160% (10 µg/mL) after the mustard plants were irrigated with the water containing MWCNTs [191]. According to TEM observations, MWCNTs can infiltrate cells in adult plants, with increased accumulation under salt stress. The positive impacts of MWCNTs on growth in NaCl-treated plants include increased water uptake, supported by more favorable energetic forces driving this process, and improved net CO_2_ assimilation. In comparison to salt-stressed plants, MWCNTs caused alterations in the lipid content, flexibility, and permeability of the root plasma membranes. Additionally, there was an increase in aquaporin transduction, which facilitated water intake and transport, thereby reducing the detrimental consequences of salt stress (Fig. 7C) [54]. Owing to its unique spectroscopic characteristics, Raman spectroscopy is one of the most sensitive methods for the non-destructive analysis of the presence of CNTs in plant organs and cells. The presence of clustered CNTs in the flower structures was demonstrated by Raman spectroscopy analysis (Fig. 7D) [53].

In hydroponic conditions, CNTs can enter various crops such as the seeds of cabbage, [54] rice, [221] tomato, [130, 222] soybean, [148] and maize [223]. CNTs may behave differently in natural environments depending on their size (length and diameter) and functionalization and the environmental conditions [224]. Several studies have demonstrated that functionalized CNTs seem to infiltrate plants more easily than non-functionalized CNTs. The amount of chemicals accumulated varies greatly depending on the crop species, as well as the type and concentration of the CBN used. CNTs improve morphological development and biomass in the leaves, stems, and roots by positively regulating the genes involved in leaf and root growth, and by increasing the auxin concentration, resulting in positive impacts on plant growth [225]. Functionalized CNTs directly enter cells with the help of biomacromolecules such as proteins, antibodies, or DNA on the surface of CNTs, which is associated with a mechanism of energy-dependent endocytic uptake [130, 148, 191, 222]. Both functionalized and non-functionalized CNTs can penetrate the cell when contamination occurs in the root. These CNTs can then be transported to the upper parts of plants and are distributed via transpiration by sharing the vascular system with water and nutrients. Low concentrations of CNTs were detected in the stems, shoots, leaves, and fruits of plants [150, 221, 226–229]. The uptake process of SWCNTs into plant cells has been demonstrated in plant cell cultures. Water-soluble SWCNTs with a length of < 500 nm were found to be responsible for the intracellular penetration of tobacco (*N. tabacum*) cell culture [43]. Although nanotube penetration was limited in the presence of endocytosis inhibitors, the SWCNTs could penetrate both the hard cell wall and the cell membrane, which was most likely mediated by fluidic-phase endocytosis. SWCNT uptake into *A. thaliana* mesophyll cells was unaffected by temperature, suggesting that the uptake occurs via a non-energy–dependent endocytosis mechanism [230]. Treatment of SWCNTs (5–30 μm) caused endocytosis-like structures to develop in the membranes of *A. thaliana* leaf cells. Internal penetration of functionalized magnetic SWCNTs of carrot cells and canola was demonstrated using external magnetic forces, indicating the potential of SWCNTs to serve as biomolecule delivery carriers in the future [231].

MWCNTs were found in the cells of plant seeds and seedlings [61, 131, 148, 232]. At the cellular level, individual MWCNTs or aggregates of MWCNTs may pass through the plant cell wall, which is considered to serve as a protective barrier against external chemicals entering plant cells [49, 233]. MWCNTs and other NMs can pierce the cell membranes and are then ingested via endocytosis [43, 232]. MWCNT uptake and accumulation in *Onobrychis arenaria* seedlings were shown to change the plant’s morphology and metabolic properties. The effect of an engineered NM containing MWCNTs on the growth of *O. arenaria* seedlings (at concentrations of 100 g/mL and 1000 g/mL) enabled the nanotubes to penetrate the cell wall in the roots and translocate to the leaves [234]. Despite the fact that MWCNTs are usually larger in diameter and length than fullerenes and SWCNTs, plant uptake and internal translocation have been observed for MWCNTs. By perforating and developing new pores, MWCNTs can enter plant cells and penetrate the hard seed coverings [130]. For example, MWCNTs were found in the germinating seeds of various crops such as barley (*H. vulgare*), soybean (*G. max*), and maize (*Z. mays*), with a diameter of approximately 15–40 nm [148]. MWCNTs with a small diameter of approximately 13 nm were also able to penetrate the cell walls of wheat (*T. aestivum*) roots [151] and red spinach *(A. tricolor)* seedlings [235]. According to Wild and Jones [233] MWCNTs with a diameter of 110–170 nm were able to penetrate the epidermis of the cell wall and reach the cytoplasm of the wheat root hair up to 4 μm. The effects of long-term exposure to MWCNTs on the growth of three important crops (barley, soybean, and corn) were investigated. These crop species were grown in a hydroponic condition with MWCNTs at a concentration of 50 g/mL. After 20 weeks of consistent exposure to the MWCNTs, there were no significant negative impacts on plant development. MWCNT-exposed crops demonstrated several beneficial phenotypic changes in addition to a higher photosynthetic rate. According to Raman spectroscopy with point-by-point mapping, the MWCNTs in the hydroponic solution traveled into all evaluated species and were disseminated in the studied organs (leaves, stems, roots, and seeds), as shown in Fig. 7E, F [229]. In addition, MWCNTs were detected in xylem and phloem cells in the roots of maize and soybean plants [150]. Larue et al. [226] demonstrated that the translocation of MWCNTs (less than 0.005%) from the root to the shoot and their accumulation in plant cells do not cause any adverse effect on the development and physiology of wheat (*T. aestivum*) and rapeseed (*B. napus*), which is most likely triggered by transpiration [191]. MWCNTs have been found in the vegetative shoot organs and flowers of tomato plants [53]. Servin et al. [34] revealed that CNTs can also translocate through capillary action in plants. As CNTs reached a narrow point, they blocked the transport of nutrients and other materials in the plant. According to Zhai et al. [150], p-MWCNT, c-MWCNT, and a positively charged MWCNT were taken up in maize and soybean plants grown in hydroponic solutions containing up to 50 mg/L MWCNTs, and all three MWCNTs were translocated from the roots to the leaves. TEM observations suggested that the MWCNTs moved quickly from the stems to the leaves. Therefore, CNTs could penetrate into the roots of mature plants and transport them to the upper organs. Additionally, CNTs adsorbed compounds could be released into plants, which provide a route to effectively deliver drugs or nutrients to specific sites of intact plants. However, CNTs also aggregate within the roots, which might cause adverse effects such as inducing potential nanotoxicity, inhibiting nutrient transport and affecting plant growth. More research on nano-agriculture is needed for human health and safety, particularly in the roots of edible plants, to minimize increased exposure to pollutants and nanoparticles.

### Defense mechanism of CNTs against environmental stresses

Various biotic (pathogens and herbivores) and abiotic (salinity, drought, heat, high light, and heavy metals) stresses on crops are exacerbated by shrinking arable land, scarcity of water resources, climate change effects, and the use of low-quality agrochemicals, all of which have negative impacts on crop growth and yield. Annually, drought and salinity are responsible for billions of dollars in agricultural losses [236–238].

**Fig. 8:**
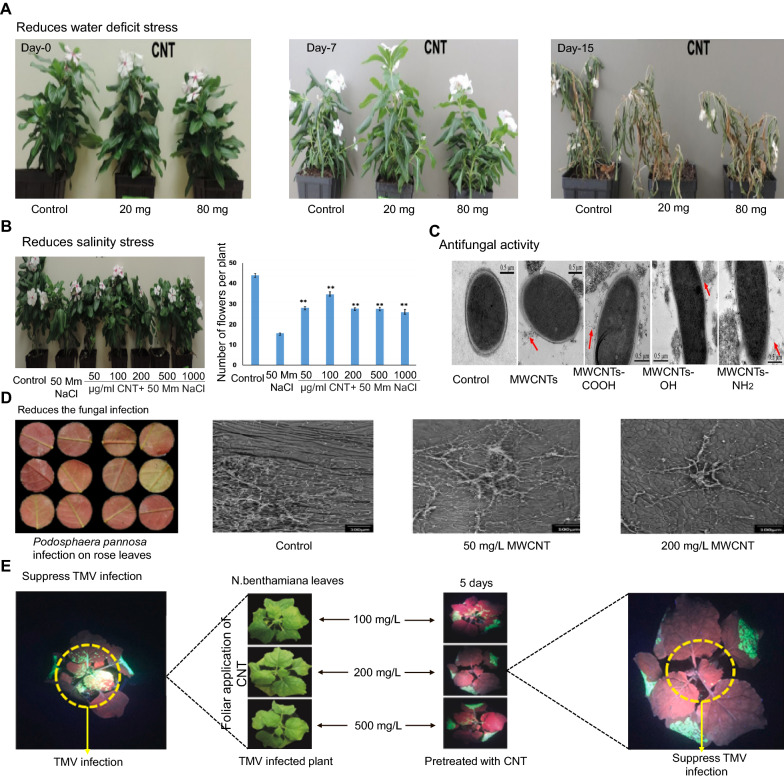
Defense mechanism of carbon nanotubes (CNTs) against environmental stresses. **A** The effect of CNTs on the phenotype of *Catharanthus* plants grown in the presence of carbon-based nanomaterials (CBNs) at day 0, 7, and 15 of water-deficit stress [134]. **B** Long-term application of CBNs to saline soil reduced salt stress toxicity and increased *Catharanthus* growth and yield. CBNs were added to saline soil and had a favorable effect on flower production in *Catharanthus*. **C** After incubation, transmission electron microscopy images of spores with deionized (DI) water and multi-walled carbon nanotubes (MWCNTs). Microscopy images of spores treated with and without (control) MWCNTs, MWCNTs-COOH, MWCNTs-OH, and MWCNTs-NH_2_. The MWCNTs around the spores and the magnified location are indicated by red arrows [242]. **D** Photographs of *Podosphaera pannosa*-infected rose leaves after exposure to 50 and 200 mg/L MWCNTs. Scanning electron microscopy images of rose leaves infected with *P. pannosa* after treatment with 50 and 200 mg/L MWCNTs [260]. **E** Foliar application of CNTs to combat tobacco mosaic virus (TMV) and develop resistance in *Nicotiana benthamiana* [181]

Several studies have demonstrated the effects of CNTs on plant growth and productivity under various abiotic stresses. Hatami et al. [134] revealed the positive effect of SWCNTs (50–800 µg/mL) on the seed germination of *Hyoscyamus niger* under different levels of drought stress (0.5–1.5 Mpa) for 14 days, demonstrating that SWCNTs at low concentrations significantly increased drought tolerance via improving water uptake and activating plant defense mechanisms such as up-regulation of starch hydrolysis processes, and reduction in oxidative damage markers (e.g., H_2_O_2_, malondialdehyde concentration) and electrolyte leakage [239]. CBNs such as CNTs and graphene were also found to reduce the adverse effects of NaCl. Long-term application of CBNs under salt stress improved the desirable phenotypic features of *Catharanthus* (higher flower number and leaf number) and cotton (increased fiber biomass). In the presence of CBNs, mature *Catharanthus* plants increased their survival without leaf wilting as compared to untreated *Catharanthus* grown under water-deficit conditions (Fig. 8A, B) [134]. Wang et al. [240] examined the antifungal activity of six CBNs (SWCNTs, MWCNTs, GO, reduced GO, C_60_, and activated carbon) against two significant plant pathogenic fungi. Among them, SWCNTs (500 mg/L) exhibited the strongest antifungal activity, followed by MWCNTs (500 mg/L), GO (500 mg/L), and reduced GO (500 mg/L), whereas the activated carbon had no antifungal activity at the tested concentration range [240]. Another study demonstrated the effect of early foliar exposure of C_60_ and CNTs (200 mg/L) against single-stranded RNA TMV. At the same time, these NMs strengthened the plant’s defense mechanism by enhancing photosynthetic performance and inducing TMV defense responses, as seen by changes in antioxidant enzymes and defense-related phytohormones (Fig. 8E) [181]. C_60_ and CNTs showed a combined impact on suppressing viral symptoms, lowering viral intensity in apical tissues, and improving photosynthetic apparatus function [181]. Hao et al. [241] showed that foliar application of 200 mg/L MWCNTs and reduced GO nanoparticles inhibited the growth of the plant pathogen *Podosphaera pannosa*, which causes powdery mildew in roses (*Rosa rugosa* Thunb.) (Fig. 8D). Surface functional groups (OH-, COOH-, and NH_2_) added to MWCNTs could expand their antifungal activities against an important plant pathogen, *Fusarium graminearum*. When this pathogen was treated with MWCNTs (500 g/mL) along with functional groups, the length of spores declined by almost half, from 54.5 μm to 28.3, 27.4, and 29.5 μm (Fig. 8C) [242].

MWCNTs could reduce the toxic effects of polyaromatic hydrocarbons on the soil microbial community. The effects of MWCNTs and fullerenes (C_60_) on pesticide accumulation in agricultural plants, including zucchini, corn, tomato, and soybean, were investigated, demonstrating a 21–80% decrease in the accumulation of weathered organochlorine pesticides such as DDx (DDT + metabolites) or chlordane in the four crops, depending on species and nanotube concentration; however, C_60_ exhibited species- and contaminants-specific effects on pesticide uptake effects, ranging from complete suppression of DDx uptake (corn/tomato) to 34.9% increase in chlordane accumulation (tomato/soybean). According to these findings pesticide accumulation varies substantially depending on crop species and carbon nanomaterial type and concentration [57]. Adsorption studies revealed that the fate and transport of adsorbed organic contaminants were affected by CNTs, which may potentially alter their bioavailability and toxicity [243]. For instance, SWCNTs showed high adsorption capacity toward phenanthrene and reduced the toxicity of the residue to algae [244]. MWCNTs can also act as pollutant carriers, controlling contaminant buildup in crops, and the enhanced impact of the contaminants is dependent on the concentration of the MWCNTs. The bioaccumulation of most contaminants increased by 10–30% (1 µg/mL) and 20–160% (10 µg/mL) in mustard plant after treatment with MWCNTs [191]. In the chloroplast, CNTs enhanced the formation of chlorophyll and carotenoids, while also acting as a carbon source to accelerate carbon fixation and speed up electron transport, resulting in improved photosynthesis [229].

CNTs can also affect plant biochemical and physiological features by altering photosynthesis and activating plant defense systems via positively regulating genes that respond to stress [245]. SWCNT-treated soybean seeds showed high resistance to drought stress via increasing the activities of catalase, superoxide dismutase, and other enzymes [246]. Well-dispersed MWCNTs functionalized with stronger functional groups resulted in better growth in tomato plants, which was mainly attributed to the activation of aquaporin [149, 222]. TEM observations revealed that MWCNTs could enter adult broccoli cells, with higher accumulation under salt stress. Increased water uptake, facilitated by more favorable energetic forces driving this process, and increased net CO_2_ assimilation were the main positive effects of MWCNTs on growth in NaCl-treated plants. In comparison to salt-stressed plants, MWCNTs caused alterations in the lipid content, stiffness, and permeability of the root plasma membranes. There was also an increase in aquaporin transduction, which facilitated water intake and transport, thereby reducing the detrimental consequences of salt stress [54].

## Challenges and future perspectives

CBNs, notably CNTs, have gained considerable attention since their discovery. Because of their unique and distinguishing physiochemical properties, including small size, large surface area, and capacity to penetrate cell walls, CNTs offer great potential for future research. Moreover, CNTs demonstrate high potential to promote agriculture sustainability by improving plant growth and development, controlling the release of nutrients and fertilizers that enhance target activity, and causing physiological changes in plants that lead to enhanced agriculture productivity under environmental stress conditions.

Despite the benefits outlined above, there are still several barriers to the application of CNT-based treatments on plant systems. Owing to their small size, large surface area, and ability to penetrate cell walls and interact with intracellular structures, CNTs can induce potential cellular and genetic phytotoxicity in plant cells by promoting oxidative stress and changing the gene expression of plants. Some studies also demonstrated that CNTs have cytotoxic effects such as reduction in cell viability, delayed flowering, reduced root length, wilting and curling of leaves, loss of pigment, yield reduction, and even cell death due to apoptosis [217, 235, 247, 248]. Furthermore, the impacts of CNTs on microbial diversity and their threat to the beneficial microbial population have also been addressed in several studies [249, 250]. CNTs could potentially build up in the soil, inhibiting the diversity and population of soil microbes. Some studies also revealed that MWCNTs can act as contaminant carriers, influencing the accumulation of contaminants in crops with different consequences depending on the concentration of contaminants adsorbed on the MWCNTs. CNTs can also penetrate the roots of mature plants, which are then translocated to the upper organs to reach the edible parts of crops [191]. The cost of CNTs is another major challenge that needs to be considered. When compared to conventional fertilizers, the cost of SWCNTs and MWCNTs is relatively high. As a result, CNTs as antimicrobial agents will only be able to compete with conventional antimicrobial products if they can be produced at a lower cost. The scalability of the synthesis of CNTs as well as their reusability must be addressed in future studies.

Currently, several researchers are focusing on the interaction of CNTs with plant systems; however, this field is still in the nascent stage, and further research is needed to understand the mechanisms that influence plant growth and toxicity. CNTs promote water uptake during seed germination in plants. However, the mechanism involved in the uptake of water inside the seed remains unknown. Hence, further investigation is required to confirm the water channel gating mechanism in plant cells. CNTs have been shown to play a role in the physiological response under abiotic stress. However, the CNT–plant cell interface under stress conditions, as well as the management of drought tolerance of valued crops in arid and semi-arid zones require further detailed investigation. SWCNTs are more likely to penetrate the plant systems and translocate to various parts of the plants. However, little information is available regarding the behavior and mechanism of translocation. The possibility of alteration in gene expression in plants because of CNTs also warrants further investigation. The safety of consuming CNT-contaminated plant organs is an important issue that needs to be further investigated, because CNT-contaminated food products can transfer CNTs to the human body through ingestion. Monoculture tests must also be conducted to assess the cytotoxic effects of CNTs on soil microorganisms. Therefore, toxicity evaluation of CNTs must be considered before they are commercially utilized for agricultural purposes, and reliable and effective techniques should be suggested to evaluate and mitigate the ecotoxicological consequences of CNTs.

## Conclusions

This review comprehensively summarizes the roles of CNTs in plant growth and development, along with the mechanisms involved in their potential application in sustainable agriculture. Because of their remarkable physiochemical properties, CNTs have sparked substantial interest in a variety of agriculture applications. The diameter and helicity of the graphene sheet as well as the number of graphene layers have a significant impact on the physicochemical properties of CNTs. As a core concept of sustainable agriculture, minimal agrochemicals should be utilized with low production costs but higher outputs. As the synthesis and utilization of CNTs in different sectors, particularly in agriculture, continues to grow, their dispersion into the environment will increase. Therefore, it is important to evaluate the behavior and impacts of CNTs on the ecosystem. To gain a better understanding of the mechanistic pathways of the absorption and distribution of CNTs in plants, more studies are urgently needed to enable the safer usage of CNTs in the development of sustainable agriculture.

## Data Availability

Not applicable.

## Abbreviations

NMs: Nanomaterials
ENMs: Engineered nanomaterials
CNTs: Carbon nanotubes
SWCNTs: Single-walled carbon nanotubes
MWCNTs: Multi-walled carbon nanotubes
TEM: Transmission electron microscopy
ROS: Reactive oxygen species
CBNs: Carbon-based nanomaterials
PVA: Polyvinyl alcohol
FITC: Fluorescein isothiocyanate
QDs: Quantum dots
TMV: *Tobacco* *mosaic* *virus*
YFP: Yellow fluorescent protein
GFP: Green fluorescent protein
